# RCBTB2 in cancer: context-dependent functions and translational boundaries

**DOI:** 10.3389/fonc.2026.1947868

**Published:** 2026-08-17

**Authors:** Chuanhai Cai, Jianjun Du, Liga Tu, Wei Li, Ren Mo

**Affiliations:** 1 Graduate School, Baotou Medical College, Inner Mongolia University of Science and Technology, Baotou, China; 2 Department of Urology, Inner Mongolia People’s Hospital, Inner Mongolia Urological Institute, Hohhot, China

**Keywords:** biomarker, copy-number alteration, molecular classification, primary cilium, RCBTB2, tumor heterogeneity

## Abstract

RCBTB2 (RCC1 and BTB domain-containing protein 2), located at chromosome 13q14, is affected by deletion, amplification, expression change, or gene fusion across the cancers studied to date. The divergent directions of these alterations do not fit a tumor-suppressor model with a fixed direction of action. Here, genetic, transcriptomic, protein-level, and functional evidence is integrated across reported cancer contexts and interpreted through three layers: the genomic neighbourhood, cell lineage, and signalling or cellular state. Within this model, the 13q14 neighbourhood determines whether RCBTB2 is altered independently or alongside genes such as RB1. Its functional consequences are further constrained by lineage-specific networks and may vary with proteostatic and ciliary states. Direct evidence connects RCBTB2 with GPAA1-related proteostasis in prostate cancer and primary ciliogenesis in mammalian cells. The proposed link between altered ciliogenesis and Hedgehog signalling in SHH medulloblastoma, however, has not been tested. On current evidence, RCBTB2 merits evaluation as a context-dependent classification or prognostic candidate, not as a broadly applicable therapeutic target. Resolving gene-specific effects within 13q14, testing cross-lineage interactions, and measuring incremental clinical value in independent cohorts are therefore the immediate priorities.

## 1 Introduction

Low-frequency candidate regulators with tissue-specific relevance continue to emerge from cancer genomic studies. Unlike canonical drivers, they may show no recurrent hotspot mutations, appearing instead through copy-number changes, altered expression, abnormal protein localization, or regional rearrangements. RCBTB2 illustrates this interpretive challenge. Its location at 13q14 and loss of heterozygosity in tumors initially prompted a tumor-suppressive interpretation based partly on domain architecture. Later multi-omics studies, however, identified alterations of different directions across cancer types (1, 3, 5–7).

The main difficulty lies in the uneven strength of the available evidence. Cohort associations, prediction-model features, single-case sequencing findings, and cellular perturbation experiments have often been placed on the same narrative plane, obscuring the distinction among association, predictive utility, and causal mechanism. Our analysis is therefore structured around three questions: where RCBTB2 is altered, what each alteration permits us to infer, and which experiments are needed to establish an interpretable biomarker or mechanism.

### 1.1 Literature search and evidence appraisal

This narrative Review used a targeted, iterative search rather than a systematic-review protocol. We searched PubMed, Crossref, publisher records, and reference lists using RCBTB2 and its historical aliases, CHC1L and RC/BTB2. These terms were combined with cancer types and with primary cilium, proteostasis, ubiquitination, and GPAA1. Searches and bibliographic checks were updated through 22 July 2026. We prioritized peer-reviewed primary studies. Reviews, classification sources, and database descriptions were retained only for background or disease-definition context. Eligible reports provided genetic, transcriptomic, protein-level, pathological, functional, or clinical information directly involving RCBTB2. Numerical claims without an identifiable primary source were not treated as established findings. The final cited evidence set comprised 28 sources, including 21 primary research reports and seven review, consensus, classification, or database sources used for context.

Evidence was appraised according to study design and permissible inference. Direct mechanistic evidence required RCBTB2 perturbation with a relevant phenotype. Rescue, domain-specific testing, biochemical interaction, or *in vivo* validation increased confidence. Clinical or molecular association evidence included cohort-level copy-number, expression, outcome, or pathological data, with independent validation weighted most strongly. Computational model inclusion, small immunohistochemical series, rare fusions, and individual cases were considered hypothesis-generating unless replicated. A mechanistic bridge denotes compatible observations from separate systems without a demonstrated causal chain. **Table 1** therefore reports the observed study design and the missing validation step rather than assigning a numerical quality score.

**Table 1:** Evidence streams, inference levels, and interpretive boundaries for RCBTB2.

Evidence stream	Representative contexts and observations	Evidence basis	Interpretive boundary	Cohort/sample characteristics
Cohort-linked functional evidence	Prostate cancer: copy-number/expression association and GPAA1 degradation (1, 2, 28)	Cohort association plus cellular and xenograft perturbation; no independent clinical validation	Supports a testable tumor-suppressive mechanism, but not independent clinical utility	259 patients and 482 tumor, benign, and germline specimens in the prostate discovery/validation study; prostate cell and xenograft models for functional follow-up
Computational or transcriptomic association	Lung adenocarcinoma, medulloblastoma, and hepatocellular carcinoma (3, 4, 8–11, 17, 18)	Discovery-stage multigene or multi-omics association; limited external validation	Nominates candidates or classifier features without establishing single-gene causality	One Xuanwei tumor-normal whole-genome pair; retrospective public transcriptomic or multi-omics datasets in lung adenocarcinoma, medulloblastoma, and hepatocellular carcinoma
Regional lesion or structural variation	RCBTB2::LPAR6 in acute lymphoblastic leukemia; 13q14 loss in lipomatous tumors and possible colorectal lesions (5–7, 12–16, 27)	Concordant regional pathology and molecular evidence in soft-tissue tumors; rare or unconfirmed elsewhere	Often reflects the RB1 neighbourhood or broader structural variation	A 21-case atypical pleomorphic lipomatous tumor series plus related pathological studies; pediatric T-ALL cohort (n=25, one RCBTB2::LPAR6 case) and individual case evidence
Protein abundance or localization	Reduced nuclear and increased membranous CHC1L/RCBTB2 staining in oral lesions (19–21)	Small immunohistochemical series; no outcome validation	Requires validated assays and independent prediction of malignant transformation	Pilot oral-lesion immunohistochemistry; OED study included 40 OED specimens and 11 normal oral mucosa controls
Mechanistic bridge	RCBTB2-dependent ciliogenesis in mammalian cells linked conceptually to Hedgehog signalling in SHH medulloblastoma (8–11, 24)	RCBTB2 perturbation with a ciliary phenotype outside medulloblastoma; no GLI or tumor test	No demonstrated RCBTB2-to-GLI-to-tumor causal chain	IMCD3 and NIH3T3 mammalian cell models for ciliogenesis; medulloblastoma linkage inferred across separate datasets and experimental systems

## 2 Evidence landscape across cancer contexts

Across cancer contexts, the RCBTB2 literature becomes more interpretable when grouped by permissible inference rather than organ site. The recurring evidence streams comprise cohort-linked functional evidence, computational associations, regional lesions, protein localization, and mechanistic bridges. Their evidential bases and corresponding boundaries are compared in **Table 1**. Of these streams, prostate cancer offers the most coherent combination of cohort and preclinical functional evidence; evidence elsewhere is predominantly associative or hypothesis-generating.

### 2.1 Cohort-linked association with functional follow-up

The most developed evidence chain for RCBTB2 currently comes from prostate cancer. Ross-Adams et al. integrated copy-number and transcriptomic data from 482 tumor, benign, and germline samples from 259 patients, associating alterations in RCBTB2 and five other genes with disease progression (1). This result places RCBTB2 within a multigene risk signature but does not identify it as an independent driver. The need for multivariable and multiregional interpretation is reinforced by analyses of genomic and microenvironmental heterogeneity (2).

Functional evidence adds a mechanistic layer to this cohort association. In prostate cancer models, RCBTB2 promotes GPAA1 degradation and suppresses cell proliferation, migration, and invasion (28). The finding directly supports a proteostasis-related role under the tested preclinical conditions, although the evidence remains limited to cellular and xenograft models. Taken together, these studies define a testable tumor-suppressive mechanism in prostate cancer without yet establishing independent prognostic value or clinical targetability.

### 2.2 Computational and transcriptomic candidate associations

The implications of computational and transcriptomic evidence differ across lung adenocarcinoma, medulloblastoma, and hepatocellular carcinoma. In each setting, RCBTB2 generally appears as one component of a broader mutation spectrum, expression signature, or multi-omics model. Its inclusion can nominate a candidate or contribute to classification, conditional on the performance of the wider feature set, but cannot by itself establish single-gene causality or clinical utility.

Whole-genome sequencing of Xuanwei lung adenocarcinoma placed RCBTB2 among candidate tumor-associated genes in an alteration spectrum associated with exposure to indoor emissions from smoky-coal combustion (3). Separately, an integrative analysis incorporated RCBTB2 into a 16-gene prognostic model using expression, methylation, and clinical features (4). Neither analysis independently validates RCBTB2 as a biomarker. The geographically and exposure-defined origin of the Xuanwei observation also limits generalization to unselected lung adenocarcinoma populations. Independent validation would require evidence for the RCBTB2 regression coefficient, incremental discrimination, calibration, and stability, with expression association distinguished from gene-dosage or functional alteration.

Within established medulloblastoma subgroup frameworks (8, 9), one retrospective bioinformatics study identified RCBTB2 among ten survival-associated genes (10), while another reported differential expression across molecular groups and possible classification value (11). These observations nominate RCBTB2 as a prognostic or classification feature within those datasets. They provide no evidence, however, that it regulates Hedgehog signalling or constitutes a dependency of the SHH subgroup.

A more specific hypothesis arises from the ciliary evidence, but the causal bridge has not been tested. RCBTB2 knockdown reduces primary cilium formation and ciliary length in mammalian cells (24), and the primary cilium serves as a platform for mammalian Hedgehog signalling. This phenotype has not been demonstrated in medulloblastoma, nor has RCBTB2 perturbation been linked directly to GLI output or SHH tumor growth. The proposed RCBTB2-cilium-Hedgehog connection therefore joins observations from separate systems. Testing it will require knockout, rescue, and domain-mutant experiments in SHH models, together with ciliary, GLI, and tumor phenotypes.

Multi-omics integration in hepatocellular carcinoma identified RCBTB2 as a candidate subtype feature (17, 18). Within that analytical setting, the evidence supports subtype association only; it does not assign RCBTB2 a direct role in metabolic reprogramming, vascular invasion, or prognosis. Independent cohorts, spatial expression analysis, and functional perturbation could distinguish a companion marker of subtype from a regulator involved in subtype formation.

### 2.3 Regional lesions and structural variation

Because RCBTB2 lies at 13q14 near RB1 and other genes, regional lesions pose a recurrent attribution problem. RCBTB2::LPAR6 transcripts have been detected in acute lymphoblastic leukemia across a pediatric T-cell cohort, a larger transcriptomic classification study, and a case report (12, 27). The available evidence is more consistent with a companion readout of broader deletion or structural variation than with an established leukemic driver. Reclassification as a driver would require evidence for the breakpoint, reading frame, protein expression, and function.

Regional loss is more consistently documented in atypical spindle cell/pleomorphic lipomatous tumors, which frequently carry co-deletion of RB1, RCBTB2, DLEU1, and ITM2B (5–7). In this context, RCBTB2 primarily marks a chromosomal neighbourhood event rather than an independently established pathogenic lesion. Combined with morphology, RB immunophenotype, and MDM2 status, the molecular finding may aid pathological interpretation, but RCBTB2 deletion alone cannot stratify clinical behavior.

Colorectal cancer exposes a different evidential boundary: previously reported numerical claims concerning RCBTB2 frameshift frequency, protein loss, and treatment response could not be traced clearly to the cited primary studies (13, 14, 16). We therefore do not treat them as established findings. Available evidence supports, at most, testing for regional 13q14 alteration or abnormal expression in defined subsets through combined sequencing, copy-number analysis, and immunohistochemistry.

### 2.4 Protein abundance and subcellular localization

Protein-level observations in oral lesions come from small immunohistochemical studies. The gene was originally cloned in 1998 as CHC1-L (chromosome condensation 1-like) because of sequence similarity to RCC1 (29). The current HGNC-approved symbol, RCBTB2, reflects its RCC1-related region and BTB/POZ domain architecture; CHC1L remains a searchable previous symbol and literature alias. Under that earlier name, reduced overall and nuclear expression together with increased plasma-membrane staining was reported in oral squamous cell carcinoma (20). Oral epithelial dysplasia showed a similar shift toward reduced nuclear and increased membranous staining (19, 21). Altered localization may therefore accompany epithelial progression, particularly among the examined samples, but neither causality nor predictive value has been established. Clinical evaluation depends on validated antibodies and scoring thresholds, reproducibility, and independent prediction of malignant transformation.

## 3 Cross-cancer integration of molecular mechanisms

### 3.1 Gene dosage and the 13q14 neighbourhood effect

Altered gene dosage is the most consistent theme in the cancer literature on RCBTB2. Deletion or reduced expression predominates in prostate and soft-tissue tumors, whereas mutation and copy-number gain may coexist in Xuanwei lung adenocarcinoma (1, 3, 5–7). These differences need not be contradictory. The broader 13q14 interval has been dissected most extensively in B-cell chronic lymphocytic leukemia and related lymphoid malignancies, where minimally deleted regions encompass the non-coding genes DLEU1 and DLEU2 and the miR-15a/miR-16–1 cluster within DLEU2 (30, 31). Functional deletion studies support a tumor-suppressive role for the DLEU2/miR-15a/miR-16–1 locus in B-cell proliferation (31), whereas the contribution of RCBTB2 has not been isolated in those models. Larger 13q14 deletions may additionally extend toward RB1. RCBTB2 should therefore be interpreted as one candidate within a historically dense critical region rather than as the default target of every 13q14 lesion. Allele-specific expression, fine-scale breakpoint mapping, and rescue experiments are needed to separate RCBTB2-specific effects from regional co-deletion.

### 3.2 Transcription, epigenetics, and protein localization

Multi-omics studies associate RCBTB2 expression with DNA methylation or tumor subtype, but do not establish promoter-methylation-driven silencing (4, 17). Altered nuclear-to-membrane localization in oral epithelial dysplasia likewise provides a mechanistic entry point, not a cause of malignant transformation (21). Future studies should measure copy number, methylation, RNA, protein abundance, and subcellular localization in the same samples. This design could connect genomic alteration to functional phenotype.

### 3.3 Proteostasis, the cell cycle, and the primary cilium

The BTB domain of RCBTB2 suggests a role in protein interactions and ubiquitination control. Many BTB-domain proteins act as substrate receptors for CUL3-RING ubiquitin ligases, but CUL3 recognition is not universal across BTB proteins and depends on additional structural determinants (32, 33). Prostate cancer models show that RCBTB2 promotes GPAA1 ubiquitination and degradation and suppresses malignant phenotypes (28). This work provides direct preclinical evidence for a role in proteostasis, but it does not establish that RCBTB2 assembles with CUL3 or identify the complete ubiquitin-ligase machinery. Testing physical association with CUL3/RBX1, dependence on cullin neddylation, and loss of GPAA1 ubiquitination after interface-directed mutation would distinguish a CUL3-adaptor model from alternative mechanisms. Separately, RCBTB2 localizes to the Golgi apparatus and centrosome, while its knockdown reduces primary cilium formation and length in mammalian cells (24). These findings establish a ciliary function outside medulloblastoma. They do not show that RCBTB2 regulates Hedgehog signalling, GLI output, or SHH-medulloblastoma growth.

### 3.4 A context-dependent model

**Figure 1:**
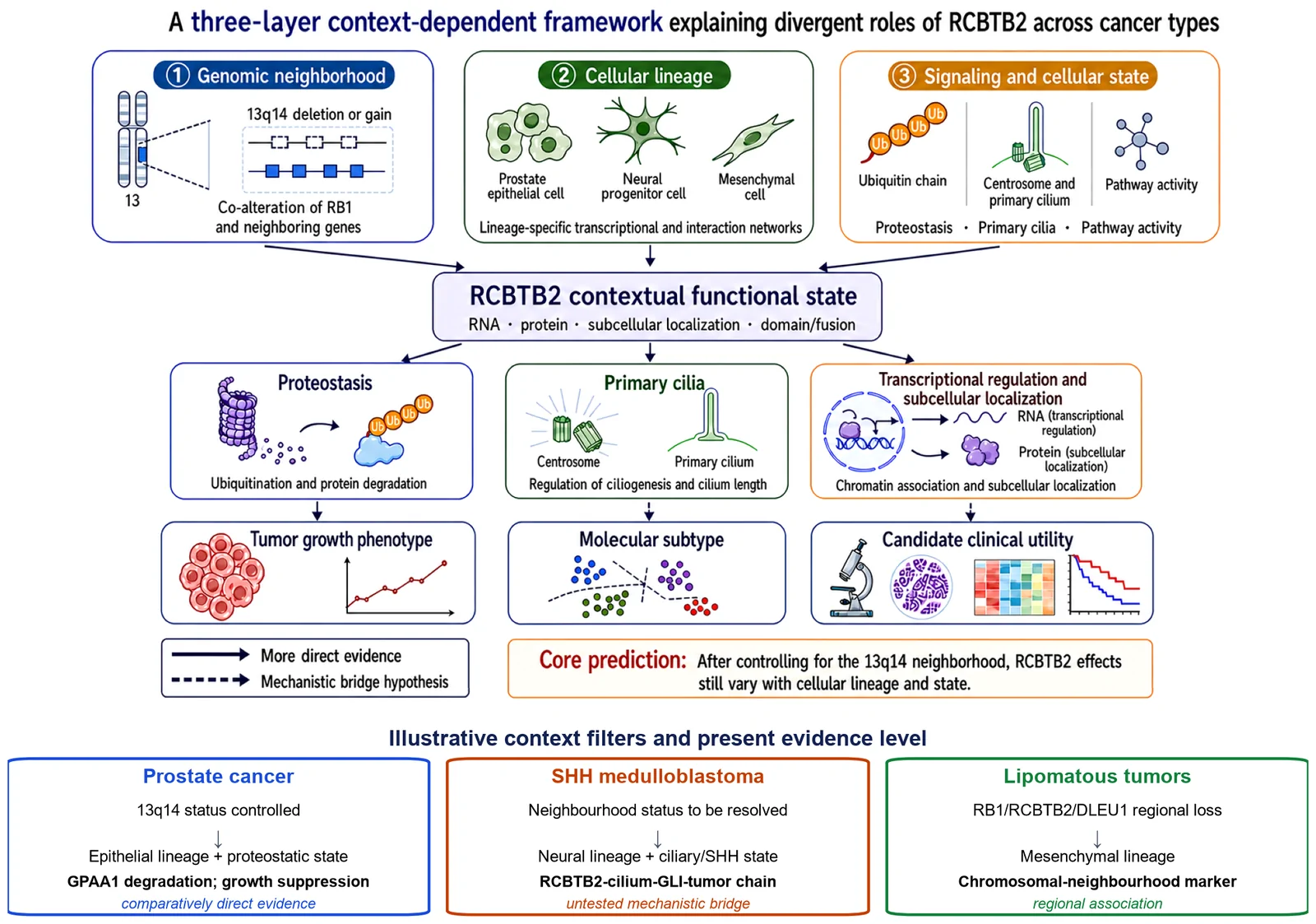
Three-layer context-dependent model of RCBTB2 function across cancer contexts. The genomic neighbourhood, cell lineage, and signalling or cellular state jointly filter the apparent functional state of RCBTB2. The context examples contrast direct prostate evidence for GPAA1-related proteostasis, regional co-deletion in lipomatous tumors, and the untested cilium-Hedgehog bridge in SHH medulloblastoma. Solid arrows denote relationships supported by comparatively direct evidence; dashed arrows denote hypothesized mechanistic bridges. The cilium-Hedgehog link is a proposed bridge rather than a demonstrated RCBTB2 pathway in SHH medulloblastoma. Experimentally, layer 1 can be uncoupled from layer 2 by selective RCBTB2 perturbation and near-endogenous rescue in isogenic models that retain the same surrounding 13q14 lesion, followed by replication of the defined perturbation across lineages. The model predicts that RCBTB2 effects will continue to vary by lineage and cellular state after the 13q14 neighbourhood is controlled.

Across the reviewed cancer contexts, RCBTB2 alteration does not correspond to a single, fixed tumor-suppressive phenotype. Instead, the evidence is consistent with a context filter whose apparent effect varies across genomic and cellular settings. We interpret this pattern through three layers: the genomic neighbourhood, cell lineage, and signalling or cellular state. The three-layer framework and its context-specific predictions are summarized in **Figure 1**. This framework is not derived from a quantitative RCBTB2-specific model; it is an organizing scheme that could also apply to other genes embedded in recurrently altered chromosomal regions. The three layers are neither assigned fixed weights nor assumed to form a universal hierarchy. Rather, their order and interaction remain empirical questions that may differ among tumor types. The genomic-neighbourhood layer determines whether RCBTB2 is altered independently or together with genes such as RB1. Lineage-specific transcriptional programs, interaction networks, and organelle dependencies then constrain the response, while proteostasis, the cell cycle, and the primary cilium may further modify it. To the extent that these layers interact, they offer a mechanism by which RCBTB2 alteration may appear as growth suppression, a risk-associated signal, or a companion readout of structural variation.

Testing the genomic-neighbourhood layer begins with allele-specific copy-number analysis, fine-scale breakpoint mapping, and explicit measurement of RB1, DLEU1, DLEU2/miR-15a/miR-16-1, and other co-altered 13q14 genes. Fine-scale CRISPR-Cas9 deletion or base-editing designs should target RCBTB2 without perturbing adjacent regulatory elements, followed by single-gene rescue at near-endogenous expression. Reciprocal rescue and combinatorial perturbation of neighbouring genes in isogenic backgrounds would test whether the phenotype is attributable to RCBTB2 alone or to the larger segment. An RCBTB2-specific effect would be supported by a phenotype after selective RCBTB2 perturbation and its reversal by RCBTB2 rescue in models matched for the surrounding lesion. Conditional on adequate control of the regional alteration, loss of that phenotype would instead identify RCBTB2 as a companion marker of the 13q14 event.

The lineage layer makes a distinct prediction: the same defined RCBTB2 alteration should produce different effects across relevant lineages under standardized conditions. This can be assessed in isogenic or otherwise matched models from at least two lineages. Evidence for lineage dependence would require a reproducible interaction between RCBTB2 status and lineage, expressed as a difference in phenotype magnitude or direction not attributable to dosage, assay platform, or baseline proliferation. Comparable effects across lineages, or disappearance of the interaction after covariate adjustment, would weigh against lineage as the principal modifier.

Cellular state can be examined within a fixed lineage and genomic background by modifying a candidate state, such as ciliogenesis or proteostatic capacity, before and after RCBTB2 perturbation. The model predicts a biologically reproducible statistical interaction in which the validated state change modifies the RCBTB2-associated phenotype. This interpretation remains conditional on adequate power and independent replication; failure to detect the interaction under those conditions would weaken the proposed state-dependent bridge.

These tests separate three evidential outcomes with different implications. Persistence after neighbourhood control supports an independent RCBTB2 effect. Dependence on lineage or cellular state identifies a context-modified mechanistic node, whereas disappearance after regional control favors a companion marker. Before any context can be described as an actionable dependency, the relevant outcome will require domain-specific rescue, orthogonal phenotypic readouts, and prespecified interaction analyses.

## 4 Biological clues beyond cancer

RCBTB2 is not restricted to cancer research. Family-based analyses of parent-of-origin effects in imprinted genomic regions reported a maternal association between an intronic RCBTB2 variant and body-composition traits (22, 23). In mesenchymal stromal/stem cells, high-resolution RNA sequencing identified RCBTB2 among eight genes consistently upregulated across species and tissue sources during cytochalasin-D-induced osteogenic differentiation (26). A prenatal chlordecone-exposure study also observed loss of Rcbtb2 expression in embryonic ovaries (25). These findings broaden the possible biological contexts of RCBTB2 to metabolism, differentiation, and reproduction, but remain associative or transcriptomic observations. They do not establish that RCBTB2 directly controls osteogenesis, adipose-bone metabolism, ovarian function, or DNA-damage repair. Keeping these clues separate from the cancer evidence avoids recasting co-occurrence across experimental systems as a unified mechanism. Within the three-layer framework, however, they reinforce the narrower proposition that lineage and cellular state can condition RCBTB2 expression or phenotype, while providing no evidence that the same downstream mechanism operates in cancer.

## 5 Clinical translational potential and its boundaries

Clinical development of RCBTB2 can be framed as a staged evaluation. Assay robustness and independent association come first, followed by tests of incremental value beyond established pathological and molecular variables. Only after these criteria are met can its ability to improve clinical decisions be assessed. Prostate copy-number and transcriptomic data and the lung adenocarcinoma multigene model provide initial settings for such evaluation (1, 4), although neither isolates the incremental contribution of RCBTB2. Relevant validation measures include discrimination, calibration, decision-curve analysis, and performance in external cohorts.

Two observations offer possible starting points for tissue-biomarker studies: regional 13q14 deletion in soft-tissue tumors and protein mislocalization in oral epithelial dysplasia (5–7, 21). Their clinical interpretation remains context dependent. Regional deletion may largely reflect alteration of the RB1 neighbourhood, whereas immunohistochemical localization varies with antibody performance, fixation, and scoring criteria. Before clinical use, assay reproducibility requires evaluation through defined sensitivity and specificity estimates and interobserver agreement in blinded multicenter cohorts.

Current evidence does not support using RCBTB2 status to select PARP, SMO, or immune-checkpoint inhibitors. It also provides no structural basis for developing an RCBTB2 agonist. A more informative route is to define functional domains, ubiquitination substrates, and lineage-specific interaction networks, then use genetic-interaction screens, organoids, and animal models to identify dependencies that are both reproducible and druggable.

## 6 Research challenges and future directions

The first challenge is evidential calibration. Large-cohort associations, single-case sequencing, prediction-model inclusion, *in vitro* functional experiments, and animal models support different levels of inference and cannot be weighted equally. Clear separation of discovery, validation, and causal evidence would improve both reviews and primary studies. A second challenge is attribution within the 13q14 neighbourhood. Fine-scale CRISPR editing, allele-specific rescue, and combinatorial perturbation of neighbouring genes offer ways to define the minimal functional unit and separate regional effects from those specific to RCBTB2.

Cross-lineage comparison provides a third priority. Measuring RCBTB2 copy number, RNA, protein abundance, subcellular localization, ciliary phenotypes, and key interacting proteins under standardized conditions could reveal whether directional differences among cancers arise from biological context or platform effects. Clinical validation raises a separate requirement for preregistered analysis plans, independent external cohorts, and comparisons of net benefit against standard clinical models. For rare fusions, interpretable reporting includes breakpoints, reading frames, protein products, and accompanying structural variants.

The most informative next steps are framed as falsifiable questions. After control of the RB1 neighbourhood, does RCBTB2 deletion still alter tumor phenotypes? In SHH-medulloblastoma models, does perturbing RCBTB2 change ciliary architecture before GLI output and tumor phenotype? Is RCBTB2-associated proteostasis reproducible across prostate cancer models? Direct experiments addressing these questions can distinguish an association marker from a context-specific mechanistic node.

## 7 Conclusions

Rather than fitting a uniform tumor-suppressor category, RCBTB2 offers a tractable model of context-dependent gene function. Across the reviewed cancers, the direction of its associated phenotypes appears to be shaped jointly by the 13q14 neighbourhood, cell lineage, and proteostatic or ciliary state. The strongest preclinical mechanism comes from prostate cancer, where RCBTB2 is linked to GPAA1-related proteostasis. Evidence in other settings is mainly associative or reflects regional deletion, structural variation, or protein localization. Although RCBTB2 supports primary ciliogenesis in mammalian cells, a connection to Hedgehog signalling in SHH medulloblastoma has not been tested. Its present translational value therefore lies in evaluation as a context-dependent candidate biomarker, not a uniform therapeutic target. Cross-lineage models, direct tests of the ciliary bridge, and independent clinical validation will determine whether RCBTB2 acts as a companion marker, a mechanistic node, or an entry point to a druggable dependency.
